# Supporting the Mental Health Needs of Military Partners Through the Together Webinar Program: Pilot Randomized Controlled Trial

**DOI:** 10.2196/25622

**Published:** 2021-10-12

**Authors:** Laura Josephine Hendrikx, Dominic Murphy

**Affiliations:** 1 Combat Stress Leatherhead United Kingdom; 2 King's Centre for Military Health Research King's College London London United Kingdom

**Keywords:** mental health support, online group-based support, military partners

## Abstract

**Background:**

Despite an increased risk of psychological difficulties, there remains a lack of evidence-based support for the mental health needs of military partners.

**Objective:**

This study aims to investigate whether the Together Webinar Programme (TTP-Webinar), a 6-week structured, remote access group intervention would reduce military partners’ experience of common mental health difficulties and secondary trauma symptoms.

**Methods:**

A pilot randomized controlled trial was used to compare the TTP-Webinar intervention with a waitlist control. The sample was UK treatment-seeking veterans engaged in a mental health charity. A total of 196 military partners (1 male and 195 females; aged mean 42.28, SD 10.82 years) were randomly allocated to the intervention (*n*=97) or waitlist (*n*=99) condition. Outcome measures were self-reported measures of common mental health difficulties, secondary trauma symptoms, and overall quality of life rating.

**Results:**

Compared with the waitlist, military partners in the TTP-Webinar had reduced common mental health difficulties (*P*=.02) and secondary trauma symptoms (*P*=.001). However, there was no difference in quality-of-life ratings (*P*=.06).

**Conclusions:**

The results suggest that TTP-Webinar is an effective intervention to support the mental health difficulties of military partners. This study provides promising evidence that webinars may be an appropriate platform for providing group-based support.

**Trial Registration:**

ClinicalTrials.gov NCT05013398; https://clinicaltrials.gov/ct2/show/NCT05013398

## Introduction

Military partners are at an increased risk of developing psychological difficulties, including problematic alcohol use, depression, anxiety, and symptoms resembling posttraumatic stress disorder (PTSD) [1-4]. This risk may partially be understood in the context that many military partners adopt a caregiving role that supports veterans’ mental and physical health difficulties [2]. Military partners report increased feelings of isolation, emotional pressure, and relationship inequality [5], and may perceive limited opportunities to develop their own self-identity within the context of the romantic relationship [6]. Furthermore, many adopt a sense of responsibility to manage stressors that may trigger veterans’ PTSD symptoms [7]. Findings suggest that this population may experience greater distress than the public (44.9%) and other caregivers (eg, 29.5% among dementia caregivers) [8,9] and that such increased burden may increase their own risk of developing health difficulties [3]. Being exposed to the adverse details of veteran military experiences, many partners may also go on to develop symptoms typical of PTSD (*secondary traumatization*) [4]. Finally, additional stressors such as employment status, ex-military status, length of veteran deployment, and veteran treatment stage may also impact military partners’ well-being [1,8,10]. Despite the clear need for appropriate support for military partners, there remains a lack of evidence-based treatment for this population’s specific mental health needs. Much of the support available for military partners is typically offered in the context of the family unit or in tandem with the veteran [11,12]. Although such interventions do reveal positive improvements in partner well-being [13], they may overlook the unique challenges faced by military partners. A recent review highlighted that there remains a paucity of programs that specifically and appropriately target military partners and their vulnerabilities [13].

Military partners face a range of barriers that may prevent them from seeking or engaging with psychological support, which may contribute to the disparity between the number of those in need of support and those who report being able to access and engage with such support [1,14]. Military families often adopt the concept of toughness and self-reliance promoted in military culture [15], and partners’ negative beliefs about support-seeking behaviors and associated feelings of shame may discourage them from seeking support [6,16]. Many partners report concerns that others do not understand the difficulties they face [1]. They may also avoid seeking support for themselves in an attempt to protect veterans from being identified as having psychological difficulties, which in turn may further heighten their own distress [17]. Such concerns reflect the unique challenges faced in military relationships as compared with civilian counterparts. In addition to such stigma-related barriers, military partners face practical barriers to seeking support. Many partners assume responsibility as the family’s main financial provider, as chronic psychological difficulties often make it difficult for veterans to maintain a permanent job [6]. In addition to being an additional stressor, this introduces restrictions in partners’ time availability, and concerns that requesting time off from work would threaten their job security may prevent them from engaging with support [16]. Similarly, many military partners may also adopt the main caregiving role for children, as veterans with PTSD may demonstrate violent behavior and respond in an aggressive manner within the family home [18]. Veterans’ symptoms of PTSD and lack of interest in maintaining social connections may result in partners becoming increasingly isolated from friends and family [19], and they may end up supporting veterans in the absence of any psychological or social support to manage their own distress. Clearly, it is essential to consider how to make evidence-based treatment most accessible to military partners.

The Together Programme (TTP) is a 5-week community-based intervention that was developed to support the mental health needs of partners living alongside veterans with PTSD and other mental health difficulties. TTP is a manualized psychoeducational intervention that aims to provide military partners with an understanding of the mental health difficulties that arise following trauma and to equip them with the practical tools to empower them in supporting the veteran’s management of their symptoms while ensuring their own well-being. When piloted across 9 UK locations, TTP demonstrated promising reductions in military partners’ mental health difficulties and secondary trauma symptoms [16]. However, this study revealed that many partners were unable to engage with support because of work responsibilities, a lack of flexibility in working hours, childcare responsibilities, and issues regarding traveling distance to the venue [16]. In an attempt to increase the accessibility of mental health support for military partners, TTP was adapted into a web-based 6-week webinar intervention named the Together Webinar Programme (TTP-Webinar). Previous research comparing web-based and face-to-face support within military contexts has demonstrated similar levels of efficacy and acceptability, as well as potentially lower rates of attrition, among web-based modalities [20-22]. This study is a randomized, waitlist-controlled pilot trial that examines the effectiveness of TTP-Webinar in supporting the mental health needs of military partners. It was hypothesized that the TTP-Webinar would result in significant reductions in general psychological distress and secondary trauma symptoms, as well as improvements in overall quality of life (QoL).

## Methods

### Design and Registration

This study is a pilot randomized controlled waitlist trial (RCT) approved by the research department at the charity through which participants were recruited. The study was not prospectively registered as it was conducted to test the feasibility of offering support to military partners via a remote access group intervention and was administered as a treatment within a mental health treatment center.

### Study Recruitment

The sample of this study is partners of veterans experiencing PTSD or other mental health difficulties. Participants were recruited by writing to veterans who had engaged with Combat Stress seeking support for mental health difficulties between April 2018 and April 2019. Combat Stress is a UK-based charity offering nationwide support for veterans with mental health difficulties and receives a high number of yearly referrals, suggesting that the current sample is likely representative of partners of veterans with mental health difficulties. A total of 2051 veterans were contacted, informed about the study, and asked for consent to reach out to their partners. Once the veterans provided consent, partners were contacted directly by a research assistant and were informed about the study. Participants were screened as eligible if they were currently in an intimate relationship with a veteran who (1) met the criteria for PTSD and (2) was previously or currently engaged with Combat Stress. Of the 285 partners who expressed interest in the study, 196 (age mean 42.28, SD 10.82 years; 195 females and 1 male) provided consent, completed baseline measures that were mailed to them, and were randomly allocated to the study conditions.

### Participants

An a priori power analysis indicated that the study required a sample size of 24 participants per condition to attain a power of 0.80 to detect a 5-point reduction on the General Health Questionnaire-12 (GHQ-12) with an SD of 6.0, assuming a standard 95% significance level. Assuming a conservative 25% dropout rate, 6 additional participants were calculated per condition, yielding a minimum sample of 60 participants.

**Figure 1:**
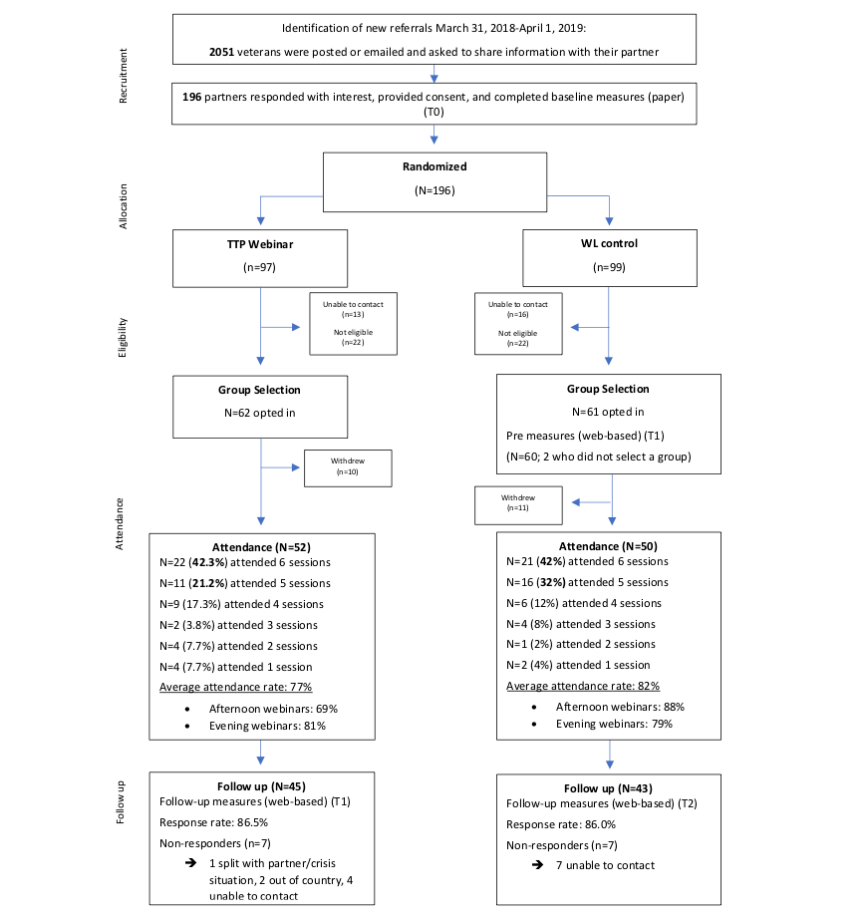
Graphical layout of participant flow in this study. TTP-Webinar: The Together Webinar Programme; WL: waitlist.

The flow of the participants in this study is described in Figure 1. A total of 196 partners provided consent, completed baseline measures, and were randomized to either the TTP-Webinar intervention (n=97) or the waitlist condition (n=99). Following randomization, 29 participants were not available, 44 were no longer eligible (eg, ended their relationship with a veteran), and 21 withdrew from the study because of difficulties with technology availability and use, previous engagement in the community TTP, childcare responsibilities, etc. As we aim to evaluate the TTP-Webinar for those who used it, these participants were excluded, and analyses included only participants who took part in at least 1 webinar session. The final sample consisted of 102 female partners (age mean 48.59, SD 10.74 years), of which 52 were randomized to the TTP-Webinar intervention and 50, to the waitlist condition. The demographic characteristics of the participants are shown in Table 1.

**Table 1:** Demographic information of participants who completed baseline measures, were randomized, and registered to a group in the TTP-Webinar intervention and waitlist condition^a,b^.

Demographics			Participant characteristics (N=102)
Age (years), mean (SD)			48.59 (10.74)
**Living with partner, n (%)**			
	Yes	90 (88.2)	
	No	10 (9.8)	
**Length of relationship (years), n (%)**			
	<9	37 (36.2)	
	>9	64 (62.7)	
**Dependents, n (%)**			
	Yes	50 (49)	
	No	50 (49)	
**Ex-military, n (%)**			
	Yes	8 (7.8)	
	No	93 (91.2)	
**Employment status, n (%)**			
	Full-time	46 (45.1)	
	Part-time	27 (26.5)	
	Not working, seeking employment	19 (18.6)	
**Level of education, n (%)**			
	Low (A levels or HNDs^c^ or NVQ^d^ or GCSEs^e^, or lower)	63 (61.8)	
	High (degree or postgraduate)	36 (35.2)	

^a^Because of missing data, numbers may not add up to the sample size and percentages may not add up to 100%.

^b^For participants who selected two responses, the average of both responses was entered. In the case of level of education and length of relationship, the highest response was considered.

^c^HND: Higher National Diploma.

^d^NVO: National Vocational Training.

^e^GCSE: General Certificate of Secondary Education.

### Procedure

Participants were informed of the study, provided consent, and returned completed baseline measures that were mailed to them. They were then randomized to the intervention or waitlist condition and rescreened for eligibility by the study coordinator via telephone.

Participants in the intervention condition were instructed to sign up to 1 of the 5 intervention groups that ran from June to July 2019. Participants in the waitlist condition were informed that they would receive details regarding the TTP-Webinar at a later point. Participants completed posttreatment measures 1 month after completion of the TTP-Webinar. To reduce nonresponse, they were sent reminder emails and, if necessary, were called by a research assistant up to three times to complete the measures via telephone. Two weeks before the waitlist groups were commenced, participants randomized to the waitlist condition were contacted via email and instructed to complete measures and sign up to 1 of the 4 waitlist groups that ran from August to September 2019.

At the end of the treatment, participants who took part in the TTP-Webinar were provided links to recordings of the 6 webinar sessions and additional self-help literature and were sent a certificate of participation if they completed the program (ie, attended at least four webinar sessions). All participants were reimbursed for their participation in a British £10 (US $13.9) Amazon voucher.

### TTP-Webinar

TTP-Webinar is a web-based adaptation of the TTP [16]. The development of TTP involved an initial review of existing programs developed to support veterans and their mental health. Two psychoeducational programs, Support and Family Education Programme [23] and Homefront Strong [24] were identified as particularly relevant. The content of the two programs was explored and adapted to meet the needs of UK partners through a process of surveying military partners to understand their needs and relevant content and format. TTP incorporates a range of techniques used in cognitive behavioral therapy (eg, to understand the maintenance of PTSD), dialectical behavioral therapy (eg, to recognize own emotions and maintain healthy boundaries), compassion focused therapy (eg, to access and develop one’s own soothing system), and acceptance and commitment therapy (eg, to help reduce avoidance behaviors and promote meaningful activity engagement).

TTP-Webinar is a live webinar intervention developed to support the mental health needs of partners living alongside veterans with PTSD and other mental health difficulties. It is a manualized program consisting of 6 hour-long weekly sessions. The content of each session encompasses a focus on (1) psychoeducation and self-management strategies for supporting veterans with PTSD or other mental health difficulties, (2) self-management strategies and skills training to enhance their own self-care, and (3) between-session homework to practice using the introduced tools. The focus of each session of the TTP-Webinar can be found in the Multimedia Appendix 1.

TTP-Webinar is delivered on a web-based platform that participants join via a link they receive. Group participants can see the facilitator and the relevant session material and presentation slides but are not able to see or hear other participants. They are encouraged to engage in the sessions by providing feedback, asking questions, and sharing their own experiences via the chat box that is viewed by all participants. During the 6-week program, participants were offered one 1:1 telephone contact, if requested, or if any risk concerns were identified.

### Outcome Measures

Demographic information was collected at baseline. Participants reported their overall QoL on a 5-point scale, ranging from 1 (*very good*) to 5 (*very bad*). Scores were reverse-scored for higher values to indicate a greater QoL.

#### Measures for GHQ-12

The GHQ-12 is a self-report measure of psychological distress within the past month [25]. It contains 6 negative (eg, *Loss of sleep over worry*) and six positive (eg, *Able to face difficulties*) items that are scored on a 4-point Likert scale ranging from 0 (*not at all or much less than usual*) to 4 (*much more than usual or more so than usual*). Positive items were reverse-scored before calculating the total score, with higher scores indicating greater psychological distress.

#### Measures for Secondary Traumatic Stress Scale

The Secondary Traumatic Stress Scale (STSS) is a self-report measure of secondary trauma symptoms within the past month [26]. The 17 items (eg, *It seems as if I am reliving the traumas experienced by my partner*) are scored on a 5-point Likert scale, ranging from 1 (*never*) to 5 (*very often*). Scores were summed to create a secondary trauma symptom score, as well as avoidance, arousal, and intrusion subscales. Higher scores indicated a greater severity of symptoms.

### Data Analysis

Missing data were inputted in a step-wise manner and were inputted only if 20% or less data on the GHQ-12 and STSS were missing (across all time points). Inputted means for missing baseline data included all 196 participants who returned baseline measures. The input means for time point 1 only included participants who took part in the program and completed measures at the given time point. The input means were computed for each condition.

Independent sample *t* tests and chi-square tests were conducted on the demographics and main outcome measures to ensure there were no differences between participants who dropped out and those who took part. Similar analyses were conducted to ensure successful randomization between the intervention and waitlist conditions.

The data were then arranged to identify the time points (T_0_ and T_1_). T_0_ ratings were used to represent the baseline measures for both conditions. T_1_ was used to represent follow-up measures for the intervention condition and preintervention measures for the waitlist condition. Next, 2 × 2 (condition: intervention vs waitlist × time: T_0_ vs T_1_) repeated measures analysis of variance (RM-ANOVA) was conducted on the GHQ-12, STSS, and QoL scores, separately. Significant interactions were followed up using paired-sample *t* tests. Additional RM-ANOVAs were then conducted to explore potential differences in the avoidance, arousal, and intrusion STSS subscales.

## Results

### Dropout

Participants who opted out of the study did not significantly differ from those who took part in terms of common mental health difficulties (*t_194_*=1.62; *P*=.11; *d*=0.23) or secondary trauma symptoms (*t_194_*=1.10; *P*=.27; *d*=0.16). However, those who dropped out reported significantly poorer QoL (mean 2.97, SD 0.82) than those who opted in (mean 3.27, SD 0.83; *t_194_*=2.47; *P*=.01; *d*=0.35). In terms of demographic differences, significant differences emerged in terms of education level (χ^2^_1_=9.5; *P*=.002; φ=0.022) and employment status (χ^2^_2_=6.5; *P*=.04; V=0.08). Although significant, further testing suggested that these differences were modest.

Additional analyses exploring differences in sociodemographic and military factors and mental health outcomes between those who dropped out of the intervention and waitlist condition are shown in Table S1 Multimedia Appendix 1).

### Randomization

Table 2 demonstrates the demographics and mental health outcomes of the intervention and waitlist conditions. As can be noted, significant differences between the 2 groups were only observed in terms of education level (*P*=.03), with a larger proportion of those in the intervention condition reporting lower educational achievement compared with the waitlist condition.

**Table 2:** Randomization of participants across the intervention and waitlist conditions^a^.

Demographics				Intervention (n=52)		Waitlist (n=50)		*P* value	
Age (years), mean (SD)				49.37 (11.08)		47.78 (10.43)		.46	
**Living with partner, n (%)**									.46
	Yes			47 (90.4)		43 (86.0)			
	No			4 (7.7)		6 (12.0)			
**Dependents, n (%)**									.23
	Yes			23 (44.2)		27 (54.0)			
	No			29 (55.8)		21 (42.0)			
**Length of relationship (years), n (%)**									.98
	<9			19 (36.5)		18 (36.0)			
	>9			33 (63.5)		31 (62.0)			
**Ex-military, n (%)**									.93
	Yes			4 (7.7)		4 (8.0)			
	No			48 (92.3)		45 (90.0)			
**Employment status, n (%)**									.42
	Full-time			22 (42/3)		24 (48.0)			
	Part-time			12 (23.1)		15 (30.0)			
	Not working, seeking employment			12 (23.1)		7 (14.0)			
**Level of education, n (%)**									.03^b^
	Low (A levels or HNDs^b^ or NVQ^c^ or GCSEs^d^ or lower)			37 (71.1)		26 (52.0)			
	High (degree or postgraduate)			13 (25.0)		23 (46.0)			
**Mental health outcomes, mean (SD)**									
	QoL^e^			3.19 (0.79)		3.35 (0.88)		.36	
	GHQ-12^f^			19.68 (6.33)		18.00 (6.60)		.19	
	**STSS^g^**			47.51 (12.57)		45.40 (14.02)		.43	
		Avoidance	19.21 (5.06)		17.71 (6.56)		.20		
		Arousal	15.62 (4.33)		15.22 (4.33)		.65		
		Intrusions	12.68 (4.68)		12.47 (4.16)		.81		

^a^Gender is not presented in the table as all participants were female.

^b^HND: Higher National Diploma.

^c^NVO: National Vocational Training.

^d^GCSE: General Certificate of Secondary Education.

^e^QoL: Quality of Life.

^f^GHQ-12: General Health Questionnaire-12.

^g^STSS: Secondary Traumatic Stress Scale.

### Outcome Measures

#### Measures for GHQ-12

**Figure 2:**
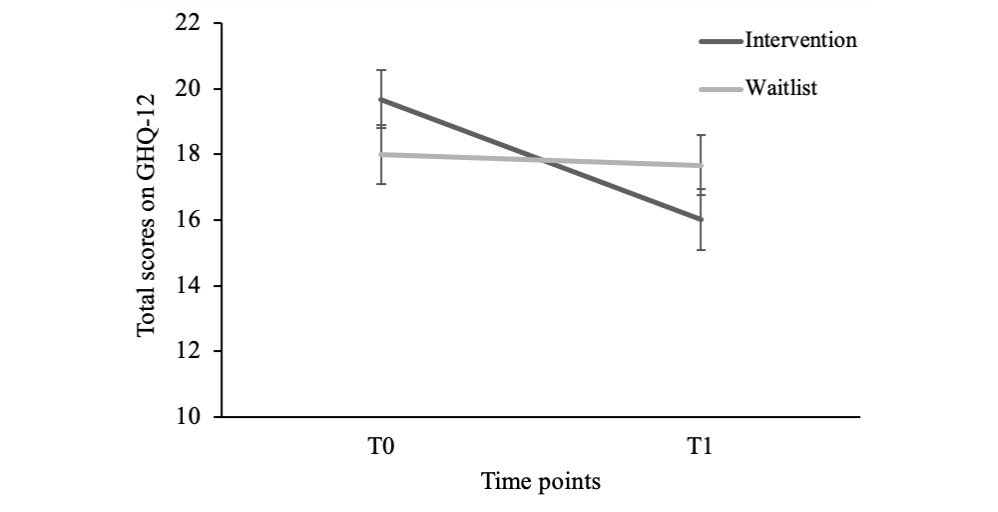
Results of a repeated measures analysis of variance of mean general psychological distress ratings of military partners, per condition (2019). Error bars represent the SEs. GHQ-12: General Health Questionnaire-12.

There was a main effect of time (*F*_1,93_=9.10; *P*=.003; *η_p_^2^*=.09) but not of condition (*F*_1,93_=0.00; *P*=.96; *η_p_^2^*=.00). There was also a significant time × condition interaction (*F*_1,93_=6.15; *P*=.02; *η_p_^2^*=.06; Figure 2). Further analyses revealed that general psychological distress was reduced in the intervention (*t_44_*=3.50; *P*=.001; *d*=0.52) but not the waitlist condition (*t_49_*=0.42; *P*=.67; *d*=0.06).

#### Measures for STSS

**Figure 3:**
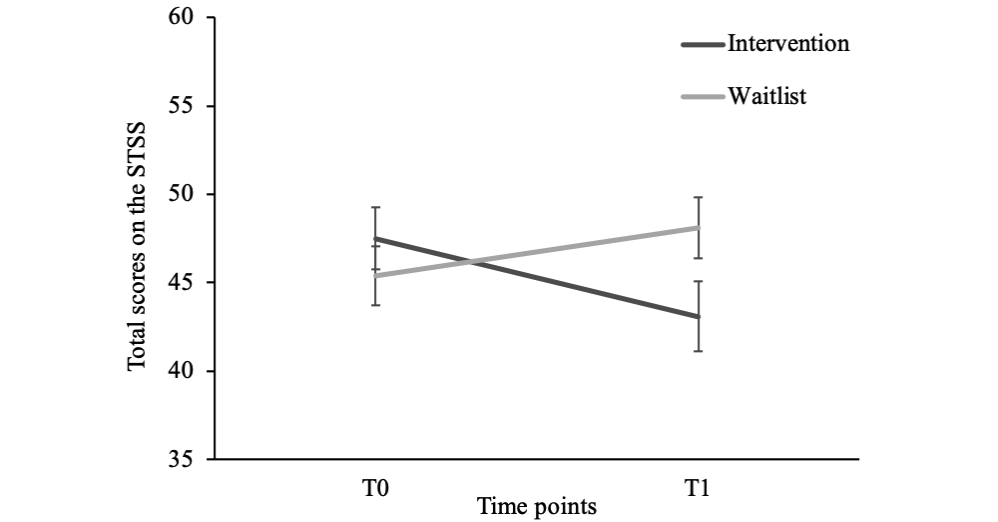
Results of a repeated measures analysis of variance of mean secondary trauma symptom scores of military partners, per condition (2019). Error bars represent the SEs. STSS: Secondary Traumatic Stress Scale.

There was no main effect of time (*F*_1,93_=1.56; *P*=.22; *η_p_^2^*=.02) nor condition (*F*_1,93_=0.20; *P*=.66; *η_p_^2^*=.002). However, there was a significant time × condition interaction (*F*_1,93_=12.56; *P*=.001; *η_p_^2^*=.12; Figure 3). Further analyses revealed that secondary trauma symptoms decreased in the intervention (*t_44_*=3.04; *P*=.004; *d*=0.45) but not in the waitlist condition (*t_49_*=-1.82; *P*=.07; *d*=0.26).

Exploratory analyses of the STSS subscales demonstrated a significant increase in intrusion symptoms in the waitlist condition (t_49_=-2.09; *P*=.03; *d*=0.30) and a decrease in both avoidance (t_44_=3.65; *P*=.001; *d*=0.54) and arousal (t_44_=2.05; *P*=.047; *d*=0.31) in the intervention condition.

#### Measures for QoL

There was no main effect of time (*F*_1,93_=1.18; *P*=.28; *η_p_^2^*=.01) nor condition (*F*_1,93_=0.13; *P*=.72; *η_p_^2^*=.00). Furthermore, the time × condition interaction was not significant (*F*_1,93_=3.45; *P*=.07; *η_p_^2^*=.04).

## Discussion

### Principal Findings

This study is one of the first pilot RCTs evaluating a psychoeducational web-based group intervention developed specifically for military partners. This study aimed to examine the impact of TTP-Webinar and to determine the feasibility of offering support via a remote access web-based platform. The findings of this study revealed that partners in the TTP-Webinar intervention condition demonstrated moderate reductions in self-reported general psychological distress and secondary trauma symptoms. However, similar reductions were not observed in self-reported QoL. One potential explanation for this may be that QoL is a complex concept and there may not have been sufficient content validity to identify changes with a single-item measure. Nonetheless, the findings provide promising initial evidence that TTP-Webinar may be an effective, web-based, structured group intervention to support the specific mental health needs of partners of veterans with PTSD and other mental health difficulties.

Although limited, previous research that has investigated support specifically aimed at military partners has focused on the effect of peer support groups on family adaptation [27], or psychoeducational groups on partners’ understanding of PTSD and self-reported behaviors of encouraging veterans to seek treatment [28]. Although the mechanisms of change cannot be established in this pilot study, numerous mechanisms are likely involved. One potential mechanism of TTP-Webinar may be the psychoeducational focus of the program. Psychoeducation for families of veterans who are experiencing PTSD and other severe mental health difficulties is a common practice within US Veteran Affairs medical centers [29]. In line with such efforts, this study extends the field by demonstrating that enhancing partner understanding of posttrauma difficulties may result in better mental health outcomes for military partners themselves. In addition to psychoeducation of veteran posttrauma difficulties, the benefits of the program may relate to psychoeducation focusing specifically on helping partners understand and manage their own mental health separate from the well-being of their veteran partner. A second potential mechanism of TTP-Webinar may be the group setting of the program. Previous research has demonstrated the benefits of group therapy for military partners [27]. Many partners experience increased social isolation and resist seeking support because of concerns that others may not be able to understand their unique difficulties [1,19]. TTP-Webinar differs from more traditional forms of group therapy, given the web, remote access delivery, and absence of participant-to-participant interaction. Nonetheless, it remains plausible that the opportunity for normalization of one’s own difficulties because of the group format and interactive platform may partially explain the beneficial effects of TTP-Webinar. Qualitative analyses of participants’ experience of TTP-Webinar indicated that psychoeducation and normalization through connecting with other military partners were key aspects of the acceptability of the program [30]. Such findings suggest that the power to develop an understanding of posttrauma difficulties and connecting to other military partners may be offered to partners through the remote, webinar program.

In addition to the importance of TTP-Webinar for supporting military partners with their mental health difficulties, the wider benefits can also be seen when considering that partner distress and poorer family functioning may result in poorer outcomes for veterans undergoing PTSD treatment [31,32]. Research suggests that a lack of partner engagement in the treatment of veterans’ PTSD treatment may have negative effects on treatment outcomes [33]. In this regard, the psychoeducational component of TTP-Webinar may be beneficial for equipping military partners with the relevant knowledge and skills to support veterans during their treatment. However, it is important to remain cautious in encouraging military partners’ engagement in veteran treatment, as it may increase the level of partner burden [34]. It is worth further investigation to determine the point at which it is most appropriate to offer military partners such a program to ensure that they are able to look after their own well-being adjunct to potentially supporting veterans’ treatment.

Military partners may develop PTSD-like symptoms that cluster in a similar manner to PTSD (avoidance, hyperarousal, and reexperiencing) because of vicariously experiencing veterans’ traumas and by taking on veterans’ feelings and experiences while trying to support them [35,36]. However, there remains a lack of clarity regarding the effects of such symptoms on military partners’ well-being, as well as a lack of investigation on how to support partners experiencing these difficulties. Exploratory analyses in this study yielded positive findings demonstrating that TTP-Webinar may be useful in attenuating partners’ avoidance and hyperarousal but not reexperiencing symptoms. The psychoeducational material delivered in TTP-Webinar focuses on enhancing the understanding of the symptoms of PTSD and depression and promoting engagement with strategies to manage such symptoms, which may explain the observed reduction in avoidance and hyperarousal. Being the first study to investigate the attenuation of secondary PTSD symptom clusters, further research is necessary to develop further insight.

### Strengths and Limitations

TTP-Webinar is a structured, manualized program developed specifically for military partners. As such, it may be argued that they have high treatment fidelity and are likely to produce similar outcomes upon replication. In line with previous evidence of good follow-up of web-delivered interventions [37], there was a high level of engagement and completion among partners who enrolled and took part in the program. The completion rates of TTP-Webinar are particularly favorable when compared with difficulties in participant retention of longer programs [38]. As military partners tend to face a complex set of demands, the beneficial findings of a short 6-week program provide further support for the appropriateness of such an intervention. Finally, this study was an RCT and thus provides strong evidence of the effectiveness of TTP-Webinar.

Despite these promising findings, this study had a few limitations. Participants were recruited via the consent of veterans. There remains a lack of clarity regarding veterans’ attitudes of their partners receiving support, and it remains plausible that veterans may withhold study information or otherwise restrict their partners’ engagement. Further dissemination of TTP-Webinar should involve consideration of how to contact military partners to promote the likelihood of engaging. Another limitation is the high dropout rate of partners who did not participate in the study because they were no longer eligible or able to be contacted. Those who dropped out differed in terms of education level, employment status, and QoL. No differences were observed between those who dropped out of the intervention or waitlist condition (Supplementary Table 1). Further dissemination of TTP-Webinar should consider how to further increase accessibility and engagement. The study was also limited in that participants were not screened for mental health difficulties. However, as the study was presented to potential participants with the aim of reducing mental health difficulties, it may be assumed that participants (subjectively) experienced distress and thus expressed interest in taking part in the study. Furthermore, the sample of this study is homogeneous, and it remains unclear whether the findings are generalizable to other groups such as male partners and partners in nonheterosexual relationships.

The smaller limitations of this study include that inferences about the long-term effects of TTP-Webinar cannot be made because of the single follow-up time point. Furthermore, in an attempt to reduce dropout, not all participants completed the follow-up measures at the 1-month interval, and some participants may have completed the questionnaires via telephone rather than on the web. It is also important to note that the webinar facilitator and research assistant involved in data collection were not blinded to the condition. However, we do not believe that such issues undermine the positive findings observed. A final limitation is that some participants may have received additional 1:1 support from the TTP-Webinar facilitator if it was requested or if any risk concerns were identified. Future replications of the study may wish to provide all partners with one 1:1 telephone call to allow appropriate risk monitoring and to ensure similar levels of support across participants.

### Conclusions

Despite these limitations, the RCT provides tentative support that TTP-Webinar is likely to be an effective, standardized program to support the psychological needs of military partners in terms of general psychological distress and secondary trauma symptoms. Furthermore, military partners are likely to find TTP-Webinar a highly acceptable program [30]. Being a web-administered program, TTP-Webinar may help to increase the accessibility of support for partners who may be unable to attend face-to-face. However, it is important to note that there were still several partners who could not engage in this study because of practical barriers, and future research should consider how to further minimize the disparity between partners in need of support and those engaging in support.

## Appendix

Multimedia Appendix 1 Webinar outline.

Multimedia Appendix 2 CONSORT-eHEALTH checklist (V 1.6.1).

## Abbreviations

GHQ-12: General Health Questionnaire-12
PTSD: posttraumatic stress disorder
RCT: randomized controlled trial
STSS: Secondary Traumatic Stress Scale
TTP: The Together Programme
TTP-Webinar: The Together Webinar Programme
QoL: quality of life
