# Supplementary material for: Supporting the Mental Health Needs of Military Partners Through the Together Webinar Program: Pilot Randomized Controlled Trial
Source: JMIR Ment Health. 2021 Oct 12;8(10):e25622. doi: 10.2196/25622 (PMC8548968; doi:10.2196/25622)
Supplement: Multimedia Appendix 1 [file mental_v8i10e25622_app1.docx]

**Supplementary material**

**TTP-Webinar outline**

TTP-Webinar is a six-week online, group-based programme that aims to provide military partner with psychoeducation regarding PTSD-related difficulties and the relational impact of being with a veteran who is experiencing such difficulties, as well as to equip them with the strategies to support their partner and take care of their own mental health needs. Each session consists of a 45-minute information giving section, followed by 15-minutes allocated for discussions and asking questions. Following each session, participants receive assigned activities to perform at home, which could involve watching online videos or listening to audio recordings, reading handouts, or going through worksheets. The content of each session is briefly described below.

**Week 1: Introduction session**

The first session covers an outline of the programme in terms of what each session will focus on, introduces participants to the eLearning resources, and informs participants of how to contact the facilitator should any difficulties arise.

**Week 2: Session 1**

This session focuses on general psychoeducation of PTSD and discusses how such difficulties can impact their romantic relationships. As homework, participants are asked to lay out goals that they would like to accomplish through the support and to read over introductory material to Cognitive Behavioural Therapy and PTSD-relevant grounding strategies.

**Week 3: Session 2**

This session focuses on psychoeducation around anger as a common symptom of PTSD as well as helps support participants to understand one’s own self-identity within the relationship and carer role. As homework, participants are asked to engage with the ‘time out’ and progressive muscle relaxation activities.

**Week 4: Session 3**

This session focuses on understanding communication difficulties that may emerge and introduces techniques about how to reconnect with partner by considering differences in communication styles. It also provides information about how to talk to others about PTSD. As homework, participants are asked to read about how to specifically talk to children and young people about PTSD and to engage with the safe place imagery activity.

**Week 5: Session 4**

This session focuses on understanding depression and introduces techniques to manage depression and other difficult emotions. As homework, participants are asked to engage with the building a compassionate image activity and to practice mindful breathing.

**Week 6: Session 5**

The final session focuses on equipping clients to understand and take care of their own needs and to give consideration to how they will be able to do so in the future. Following this session, participants are asked to reflect on the content of the webinar and to develop a wellness plan to help them in continuing to take care of their own needs.

Table S1. Differences in sociodemographic and military factors and mental health outcomes between participants who dropped out from the intervention and waitlist condition

|  | Intervention  (n = 45) | Waitlist  (n = 49) | *P* |
| --- | --- | --- | --- |
|  |  |  |  |
| **Age** | 47.98 (SD = 11.53) | 47.92 (SD = 10.49) | .98 |
| **Gender**  Male  Female | 1 (2.2%)  43 (95.6%) | 0 (0.0%)  48 (98.0%) | .29 |
| **Living with partner?**  Yes  No | 38 (84.4%)  7 (15.6%) | 42 (85.7%)  7 (14.3%) | .86 |
| **Dependants?**  Yes  No | 22 (48.9%)  22 (48.9%) | 29 (59.2%)  20 (40.8%) | .37 |
| **Length of relationship?**  < 9 years  > 9 years | 14 (31.1%)  30 (66.7%) | 17 (34.7%)  32 (65.3%) | .77 |
| **Ex-military?**  Yes  No | 3 (6.7%)  42 (93.3%) | 3 (6.1%)  44 (89.8%) | .62 |
| **Employment status**  Full-time  Part-time  Not working, seeking employment | 14 (31.1%)  13 (28.9%)  15 (33.3%) | 17 (34.7%)  9 (18.4%)  17 (34.7%) | .57 |
| **Level of education**  Low (A Levels/HNDs/NVQ/GCSEs, or lower)  High (Degree/Postgraduate) | 36 (80.0%)  9 (20.0%) | 40 (81.6%)  6 (12.2%) | .37 |
| **Mental Health Outcomes**  QoL  GHQ-12  STSS | 3.02 (SD = 0.83)  20.60 (SD = 8.05)  48.62 (SD = 14.80) | 2.94 (SD = 0.82)  20.46 (SD = 7.74)  48.64 (SD = 13.62) | .62  .93  .99 |
